# Palladium nanoparticles on InP for hydrogen detection

**DOI:** 10.1186/1556-276X-6-410

**Published:** 2011-06-02

**Authors:** Ondrej Cernohorsky, Karel Zdansky, Jiri Zavadil, Pavel Kacerovsky, Katerina Piksova

**Affiliations:** 1 Department of Physical Electronics, Czech Technical University, V Holesovickach 2, Prague, Czeck Republic; 2 Institute of Photonics and Electronics, Academy of Sciences of the Czech Republic, Chaberska 57, Prague, Czeck Republic

## Abstract

Layers of palladium (Pd) nanoparticles on indium phosphide (InP) were prepared by electrophoretic deposition from the colloid solution of Pd nanoparticles. Layers prepared by an opposite polarity of deposition showed different physical and morphological properties. Particles in solution are separated and, after deposition onto the InP surface, they form small aggregates. The size of the aggregates is dependent on the time of deposition. If the aggregates are small, the layer has no lateral conductance. Forward and reverse I-V characteristics showed a high rectification ratio with a high Schottky barrier height. The response of the structure on the presence of hydrogen was monitored.

## Introduction

An energetic barrier called the Schottky barrier is formed on the interface of a metal and semiconductor. This barrier shows a rectifying effect like classical PN diodes. This structure could be exploited as a hydrogen sensor, where the sensing mechanism consists in the change of the barrier height by the presence of hydrogen on the interface. A palladium (Pd) was used here for its ability to dissociate hydrogen molecules to single atoms. This fact is further enhanced by the nanoparticle form of Pd because of its high surface-to-volume ratio. An n-type indium phosphide (InP) played the role of the semiconductor here. Electrophoretic deposition of Pd nanoparticles from their colloid solution appeared to be the most convenient method for the interface preparation in the sense of the Schottky barrier height and, therefore, also for the sensitivity of the sensor [1].

The Schottky barrier height, *Φ*_B_, is theoretically given (in the case of n-type semiconductors) by the relation *Φ*_B _= *Φ*_M _- χ_S_, where *Φ*_M _is the work function of metal and *χ*_S _is the electronic affinity of the semiconductor. In practice, the Schottky barrier is lower than the difference *Φ*_M _- *χ*_S _(in the case of n-type semiconductors). This is caused by a Fermi level pinning explained by disorder-induced gap states - electronic states in a bandgap given by a disorder of atoms on the interface [2]. These gap states come from an imperfect metal/semiconductor interface. We can partly eliminate these unwanted gap states by methods of fabrication of the interface. It was found that the sensor has lower sensitivity when a metal is deposited by high energetic means (e.g., thermal evaporation, e-gun) [1]. A more convenient method of interface fabrication is the electrophoretic deposition of metal nanoparticles. The height of Schottky barrier prepared by electrophoretic deposition was 0.85 eV. This height is higher when compared to 829 meV reported in Ref. [1].

## Materials and methods

### Preparation of Pd nanoparticles

Pd nanoparticles were prepared in reverse micelles by a reduction of palladium(II) chloride by hydrazine according to Wu *et al. *[3]. Palladium(II) chloride, 0.05 M, and 1 M hydrazine water solutions were prepared. Equal amounts ruled by parameter *ω*_0_, defined as a ratio of molar concentration of H_2_O and AOT in final isooctane solution, *ω*_0 _= [H_2_O]/[AOT], were added to two equal amounts of 0.1 M of AOT/isooctane solution. AOT (sodium 1,4-bis(2-ethylhexoxy)-1,4-dioxobutane-2-sulfonate) plays a role of surfactant here. Parameter *ω*_0 _controls the size of the nanoparticles. Here, *ω*_0 _= 5.

In the end, these AOT/isooctane solutions, the first with palladium salt, the second with hydrazine, were mixed. The solution of Pd nanoparticles was monitored by a scanning electron microscope (SEM) (JSM-7500F, JEOL Ltd., Tokyo, Japan). The size of the particles was 10 nm with less than 10% dispersion.

### Preparation of layers of Pd nanoparticles on InP

The n-type InP wafers were purchased from Wafer Technology Ltd. (Milton Keynes). The crystallographic orientation of the wafers was 100, the carrier concentration was ≤10^16 ^cm^-3^, and the E.P.D was ≤1 × 10^5^. The full-area ohmic contact on one side of the wafer was made by an obtrusion of molten Gallium and then by the application of a conductive silver colloid paint.

Electrophoretic deposition was performed in the cell composed of two electrodes; the first is at the bottom of an insulating (Teflon) bottle, and the second is on the top. On the bottom electrode, the InP wafer was mounted by a conductive silver colloid paint, and the colloid solution was placed between the electrodes (the distance between the electrodes was 2 mm). Both polarities of deposition were realized; the negative deposition (ND), where negative voltage was applied to the bottom electrode, and the positive deposition (PD) with the positive voltage to the bottom electrode. The applied voltage was from 30 to 100 V for various times.

After the deposition, the wafer was rinsed with isopropyl alcohol in order to remove residual AOT/isooctane solution, and small contacts by colloid graphite paint were created. Current-voltage characteristics were measured using a Keithley Source-Measure Unit 237 (Keithley, Cleveland, OH, USA).

## Results and discussion

**Figure 1:**
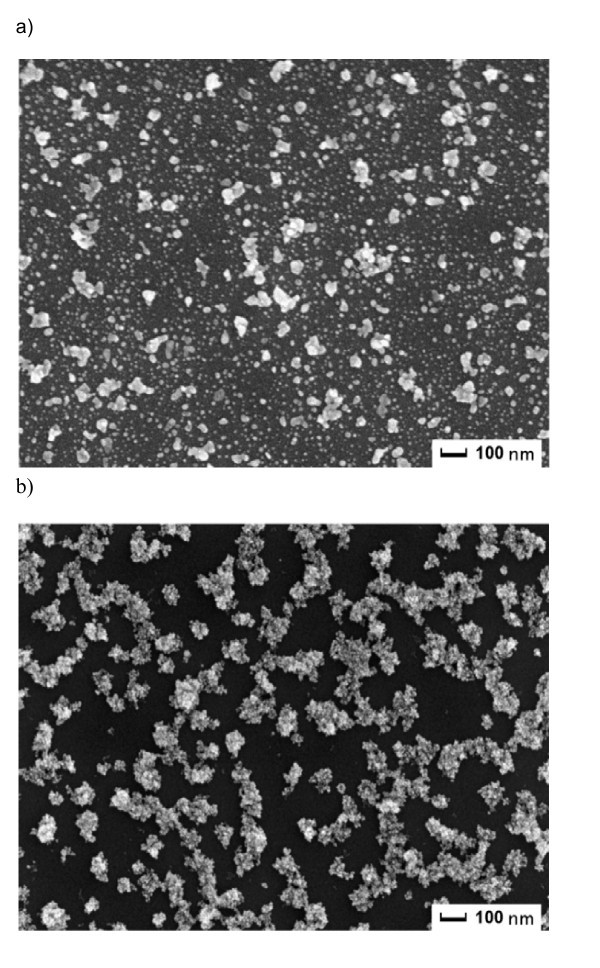
**SEM images**. (a) SEM image of PD nanolayers and (b) ND nanolayers

Secondary ion mass spectrometry (SIMS) spectra of Pd isotopes 104, 105, 106, 108, and 110 for the ND and PD layers showed that on PD layers, Pd content is about two orders of magnitude larger than in the ND layer. In Figure 1, SEM pictures of the PD and ND layers, respectively, prepared under the same conditions (66 V, 4 h), are presented. The density of the particles on the PD sample was larger while, in the case of the ND layer, small separated aggregates on the InP surface were observed. The PD sample exhibited charging in SEM; this is the reason we believe there are more nonconductive surfactant molecules which cover the majority of the InP surface. It suggests that surfactant aggregates without Pd particles are negatively charged in the solution, but the charging mechanism of nonpolar colloids is still far from clear. Because SEM observations of the particles from the colloid solution dropped on the TEM grid showed that the particles in the solution are not aggregated, it can be concluded that the particles form aggregates on InP during the electrophoretic deposition process.

An AFM profile measurement of the PD layer showed that nanoclusters on the surface are separated, and the distance between them is about 100 nm. This fact explains a significant lateral resistance when the distance between clusters is too large to enable quantum-mechanical tunneling. The size of aggregates grows with deposition time. The particles probably settle selectively in the vicinity of the previously deposited particles because of the higher gradient of the electric field. When the deposition time is long enough, e.g., 18 h, the layer turns to be laterally conductive.

**Figure 2:**
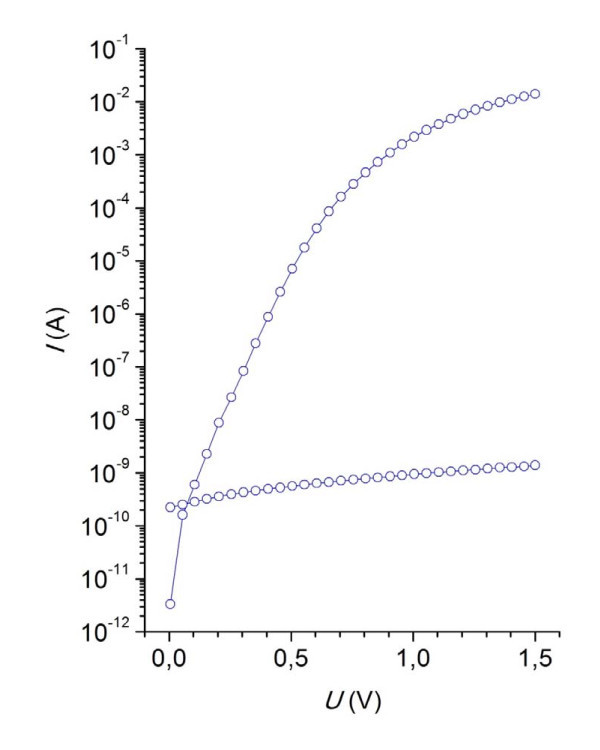
**I-V characteristics of ND Pd layers**. Higher curve is for forward bias and the lower for reverse bias.

A colloid graphite paint used for contacts makes a good Schottky barrier on an InP without a Pd layer, in contrast to formerly used colloid silver paint. I-V characteristics (Figure 2) of the ND Pd layer with graphite contact exhibit a large rectification ratio and linear part gives Schottky barrier height 0.85 eV. Although an SIMS revealed less Pd isotopes on the ND layers, they have better rectifying ratios and sensing capabilities. We suppose that this fact is due to less coverage of the InP surface in combination with the porosity and high Schottky barrier of the graphite colloid paint on the InP.

**Figure 3:**
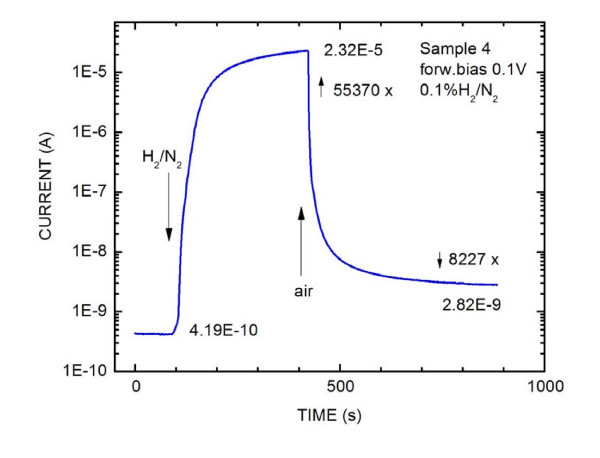
**Current-voltage characteristic**. Dependence of current on time of the ND Pd layer on InP interface in the presence of the 0.1% H_2_/N_2 _mixture. A forward bias of 0.1 V was applied.

Formerly, we assumed that the PD layers were better for sensing because of their higher content of Pd. The response of these layers, equipped with silver contacts, to the presence of hydrogen was very quick, but the current change was not very high because of the impenetrability of the silver contact for the hydrogen molecules. The current change was more than two orders of magnitude. Now, we use graphite contacts, which are porous, and ND layers with lower density of coverage, so at the places, where are no Pd nanoparticles, the graphite contact touches the surface. The response of the structure on 0.1% H_2 _in N_2 _mixture can be seen in Figure 3. The current change was of 55,000 × when the current increased from its initial value 4.2 × 10^-10 ^A to 2.3 × 10^-5 ^A. The response and recovery times are of the order of 10 s, but the full recovery to its original value, probably due to the slow release of hydrogen diffused into the crystal lattice of the Pd nanoparticles, takes about 10 h [4].

## Conclusions

Colloid solutions of the Pd nanoparticles of size 10 nm were prepared, and electrophoretic deposition of these nanoparticles onto the InP wafer was performed. The PD layers had more Pd on the semiconductor surface while the ND layers had a cleaner surface without a surplus of AOT molecules, and the surface is not fully covered. The Schottky barrier height of the ND layers was 0.85 eV. When compared to Ref. [1], the height of the Schottky barrier of the structure presented in this work was about 0.26 eV higher, and the sensing capabilities were more than 100 times better (0.1 forward voltage, 0.1% H_2_). We suppose the main improvement consist in using the ND deposition instead of the PD reported in Ref. [1] and in tuning the deposition time to prepare the appropriate porous layer. The colloid graphite also has an indispensable influence on sensing quality due to its porosity for hydrogen molecules.

## Competing interests

The authors declare that they have no competing interests.

## Authors' contributions

OC prepared Pd nanoparticles and performed SEM measurements. KZ prepared nanolayers and performed electrical measurements. JZ provided valauble consultations. PK provided material for deposition and prepared InP substrates. KP performed SEM measurements.

## References

[B1] Chou YI Chen ChM Liu WCh A new Pd-InP Schottky hydrogen sensor fabricated by electrophoretic deposition with Pd nanoparticles IEEE Electr Device L 2005 27 62 66

[B2] Hasegawa H Interface-controlled Schottky barriers on InP and related materials Solid-State Electron 1997 41 1441 1450 10.1016/S0038-1101(97)00087-7

[B3] Wu ML Chen DH Huang TC Preparation of Pd/Pt bimetallic nanoparticles in water/AOT/isooctane microemulsions J Colloid Interface Sci 2001 243 102 108 10.1006/jcis.2001.7887

[B4] Zdansky K Yatskiv R Grym J Cernohorsky O Zavadil J Kostka F Study of electrophoretic deposition of Pd metal nanoparticles on InP and GaN crystal semiconductors for H2-gas sensors Proc NANOCON 2010 B13

